# Role of Rho-Associated Protein Kinase Inhibition As Therapeutic Strategy for Parkinson’s Disease: Dopaminergic Survival and Enhanced Mitophagy

**DOI:** 10.7759/cureus.16973

**Published:** 2021-08-07

**Authors:** Huma Quadir, Knkush Hakobyan, Mrunanjali Gaddam, Ugochi Ojinnaka, Zubayer Ahmed, Amudhan Kannan, Jihan A Mostafa

**Affiliations:** 1 Internal Medicine/Family Medicine, California Institute of Behavioral Neurosciences & Psychology, Fairfield, USA; 2 Neurology, California Institute of Behavioral Neurosciences & Psychology, Fairfield, USA; 3 Diagnostic Radiology, California Institute of Behavioral Neurosciences & Psychology, Fairfield, USA; 4 Internal Medicine, California Institute of Behavioral Neurosciences & Psychology, Fairfield, USA; 5 Family Medicine, California Institute of Behavioral Neurosciences & Psychology, Fairfield, USA; 6 Medicine, Jawaharlal Institute of Postgraduate Medical Education and Research, Puducherry, IND; 7 General Surgery Research, California Institute of Behavioral Neurosciences & Psychology, Fairfield, USA; 8 Faculty Member, California Institute of Behavioral Neurosciences & Psychology, Fairfield, California, USA

**Keywords:** parkinson’s disease, mitophagy, pink1, parkin, hexokinase, rho-associated protein kinase, rock, substantia nigra, fasudil, dopaminergic survival

## Abstract

The GTP-binding protein, Rho, plays a significant role in the cellular pathology of Parkinson’s disease. The downstream effector of Rho, Rho-associated kinase (ROCK), performs several functions, including microglial inflammatory response and enhanced Parkin-mediated mitophagy. Its inhibition shows neuroprotective effects in carried studies. Parkinson’s disease pathology also rests on incomplete removal of damaged mitochondria, leading to neuronal impairment. ROCK has different isoforms, inhibition of which have been shown to decrease the adverse changes in microglia. There has also been evidence of a decreased release of inflammatory cytokines and a reduction in degradation of dopaminergic neurons on the addition of ROCK inhibitors. Additionally, ROCK inhibitors have recently been shown to increase the activity of hexokinase 2 (HK2), relocating it to mitochondria, and therefore leading to upregulated mitochondrial targeting. Understanding the cellular basis of ROCK activity and its inhibition may help us advance in creating new strategies for the treatment of Parkinson’s disease.

## Introduction and background

Parkinson’s disease (PD) is a progressive neurodegenerative disorder characterized by several neuronal symptoms, including rigidity, tremors, bradykinesia, and postural instability. Neuropathological hallmarks include loss of dopaminergic neurons in the substantia nigra, leading to decreased transmission of dopamine [1]; and intracellular inclusions containing a-synuclein [2]. PD affects one to two per 1000 of the population at any time. PD prevalence is increasing with age, with approximately 1% of the population being above 60 years of age [3]. Based on aging alone, up to 700,000 cases of Parkinson’s disease are predicted by 2040 [4]. Current PD treatments alleviate Parkinson's symptoms initially but do not prevent PD or remain effective as the disease further advances, making it essential to comprehend the molecular basis of PD.

Rho-associated kinase (ROCK) is a downstream effector of the small GTP-binding protein Rho [5]. ROCK has been demonstrated to play a critical role in the formation of reactive oxygen species, apoptosis, and chemotaxis [6]. It has been further evidenced to be responsible for the lack of neuronal regeneration [7]. Moreover, its inhibition has also been found to have antiapoptotic effects on retinal ganglion cells after optic nerve axotomy [8]. An earlier study was able to generate the neuroprotective response of pharmacological rho kinase inhibition with orally applied fasudil (ROCK inhibitor). The study discovered an amplified activation of Akt signaling after rho kinase inhibition, thus attributing rho kinase an important role in axonal integrity [9].

Additionally, there has been increasing evidence suggesting loss of mitochondrial function and its insufficient degradation as central in PD. This happens by ubiquitin ligase Parkin and protein kinase: PTEN-induced kinase 1 (PINK1) mediated mitophagy [10-11]. Amplifying this mediation of mitophagy may lead to the possible prevention of neuronal degeneration in PD. Normally, after mitochondrial damage, PINK1 accumulates on the outer mitochondrial membrane (OMM) where it phosphorylates ubiquitin, tagging the mitochondria and, in turn, recruiting the Parkin protein for mitochondrial autophagy (mitophagy) [12-13]. Another study shows that the inhibition of ROCK can amplify this pathway by increasing Hexokinase2 (HK2, positive Parkin protein regulator) recruitment to the damaged mitochondria, thereby, enhancing the targeting of mitochondria and its removal from cells [14]. However, the role of ROCK in Parkin-mediated mitophagy has not been thoroughly explored and its inhibition may have neuroprotective effects, making it of great importance to understand for future therapeutic discoveries. Here we review the evidence suggesting the role of ROCK inhibition on reducing neuronal degeneration and its enhancement of the mitophagy pathway as bases of therapeutic strategies in treating Parkinson’s disease.

## Review

Isoforms of Rho-associated kinase

**Figure 1:**
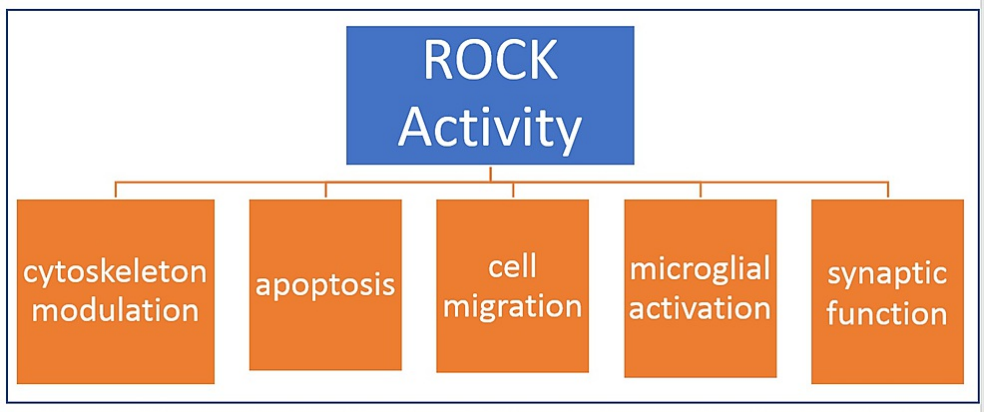
Known functions of Rho-associated protein kinase (ROCK) ROCK: Rho-associated protein kinase

ROCK is a serine-threonine molecule with diverse functions in neurons as can be seen in Figure 1. It is a regulator of actomyosin cytoskeleton organization, cell migration, apoptosis, contraction of smooth muscles cell, with a significant role seen in the pathologies of cerebral and coronary vasospasm, hypertension, and signaling in neurons. It has two homologous isoforms: ROCK1 and ROCK2 [15]. They express similar structures. ROCK1 is the more dominant of the two and is present in the lung, liver, testes, blood, and immune system. ROCK2 occupies the brain and muscles. Its activity is regulated by RhoA and RhoC, which belong to the Ras-superfamily. There has been accumulating evidence of ROCK present in increased quantities in microglial cells in Parkinson’s disease. To date, two ROCK inhibitors have been approved in Japan for clinical use. These are ripasudil and fasudil [16].

Microglial response to Rho-associated kinase activity

Microglia are phagocytic cells with migratory potential. For such cells, cytoskeletal activity is significant and not only enables them to migrate but also to remove cellular debris. This cytoskeletal activity is dependent on ROCK. Interestingly, ROCK also causes the release of cytokines and chemokines, which is initiated by ligand binding to toll-like receptors and specific receptors, releasing superoxides and nitric oxide [17]. ROCK inhibition has been shown to prevent changes in microglia in a rat model of neuropathic pain [18]. Also, Fasudil led to reduced expression of inducible nitric oxide synthase and release of proinflammatory cytokines like NO, TNF-α, IL-1β, and IL-6 [19]. Numerous studies suggest the relationship between dopaminergic neurons and microglia activation in the substantia nigra in Parkinson’s disease and increased ROCK expression in glial cells in postmortem tissue of PD patients [20-22].

Evidence of neuronal degeneration in response to Rho-associated kinase

Over the years, several studies have demonstrated neuronal cell death after induction with dopaminergic neurotoxin 1-methyl-4-phenyl-1,2,3,6-tetrahydropyridine (MPTP) and MPTP metabolite 1-methyl-4-phenylpyridinium (MPP+). Treatment of mice with MPTP, mediated degeneration of dopaminergic neurons and its terminals in the substantia nigra compacta (SNc). However, a simultaneous reduction was observed in this process on the administration of ROCK inhibitors, indicating its role in dopaminergic neuroprotection [23]. Furthermore, Fasudil (a selective RhoA/ROCK inhibitor) has demonstrated effects on the apoptosis signal-regulating kinase 1 (ASK1)/c-Jun N-terminal kinase (JNK) signal pathways, thereby demonstrating itself to be neuroprotective against apoptosis [24]. Increased ROCK activity was observed after MPTP injection with a later observation of rising dopaminergic deterioration in SNc, which suggests a major impact of ROCK activity on dopaminergic degeneration [25]. Moreover, ROCK knock-out Parkinson’s disease mouse models exhibited high dopamine levels although being subjected to MPTP as compared to wild types [26]. Interestingly, samples of the frontal cortex from atypical Parkinson’s syndrome patients (progressive supranuclear paralysis displayed high levels of RhoA effectors [27].

ROCK inhibition also suppresses the release of inflammatory cytokines (interleukin- ß and tumor necrosis factor-a) from microglial cells. The microglial inflammatory response is also mediated by the interaction of ROCK with nicotinamide adenine dinucleotide phosphate (NADPH) oxidase, producing high concentrations of reactive oxygen species to kill invading organisms and ROCK inhibitors prevent this activation [25].

Therefore, ROCK involvement in Parkinson’s disease is multifactorial, involving several pathogenic processes. It suggests ROCK inhibition being a promising candidate for dopaminergic neuron protection.

Mitophagy during normal circumstances

**Figure 2:**
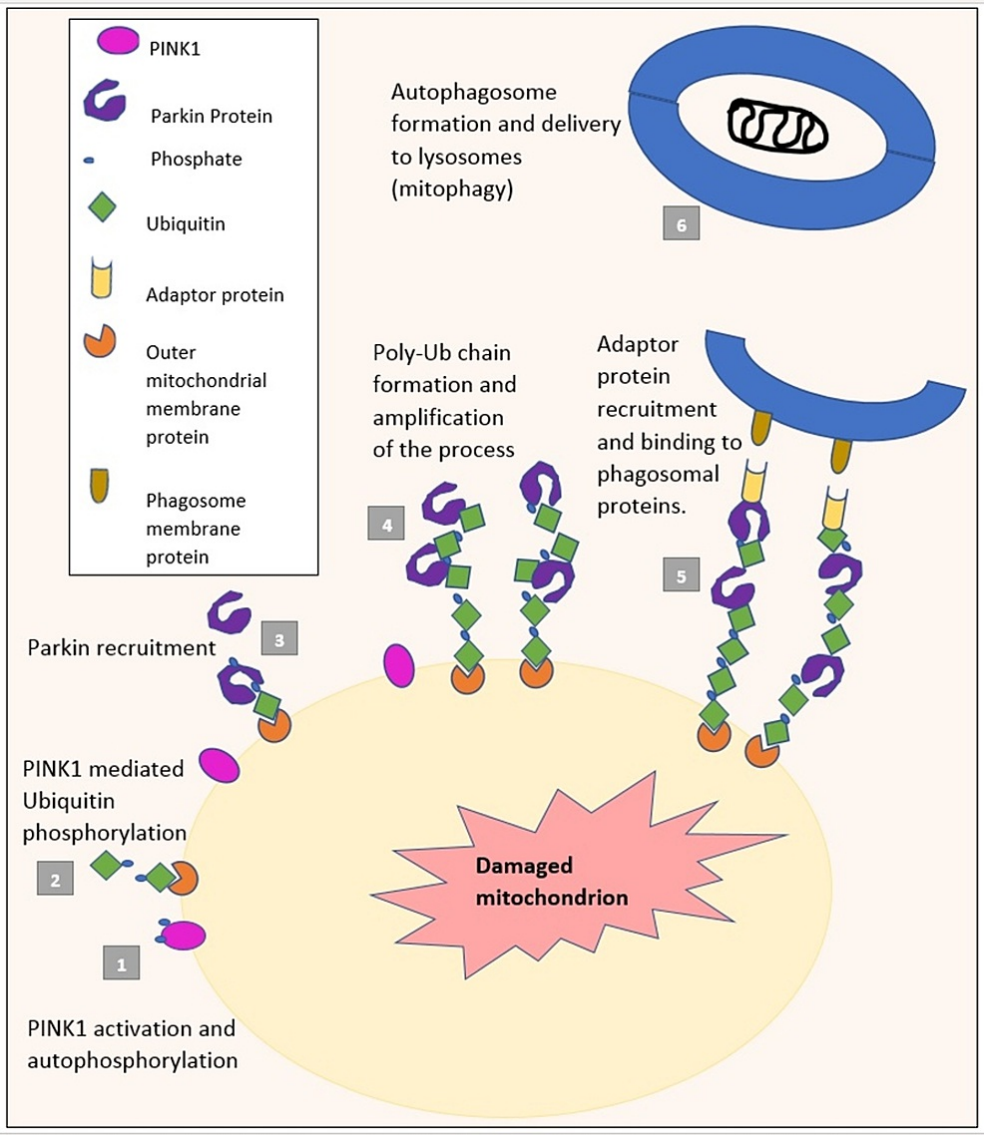
PINK1 and Parkin-mediated mitophagy PINK1: PTEN (phosphatase and tensin homolog)-induced kinase 1; Ub: Ubiquitin

Mitophagy is a complex process as seen in Figure 2. Upon damage of mitochondria or its depolarization, PINK1 settles on the outer mitochondrial membrane (OMM) and phosphorylates ubiquitin on its surface. This recruits Parkin protein. PINK1 then phosphorylates Parkin, leading to Parkin-mediated ubiquitination for attachment to the phagosome, for the process of mitophagy, which is necessary to prevent neuronal damage [28].

Interestingly, once relocating to the OMM, Parkin then ubiquitylates numerous target proteins, including HK1 and HK2, indicating a possibility of this step being required for Parkin recruitment. This reflection also states that Parkin activity may be terminated by turnover of HK1/2 after ubiquitylation. Loss of hexokinase activity would leave the PINK1/Parkin process inactive [29].

Role of ROCK inhibition in PINK1/Parkin-mediated mitophagy

As stated above, PINK1 induces the translocation of Parkin, an E3 ubiquitin ligase, to the impaired mitochondria for their removal via autophagy (mitophagy). The ROCK inhibitors are evidenced to activate the Parkin-mediated mitophagy pathway thereby causing 399 neuroprotective effects in paraquat-treated flies [14].

Studies have identified that MYO6 (myosin VI), moves towards the minus end of actin filaments, interacts with Parkin, and is recruited to the insulted mitochondria via its ubiquitin-binding domain. This myosin then initiates the formation of F-actin cages, which encapsulate the injured mitochondria [30]. F1-actin cages quarantine the damaged mitochondria from exchanging content or refusing when destined for mitophagy.

ROCK2 also has a role in phosphorylating cofilin and stabilizing the actin cytoskeleton. Therefore, a recent study has stated the possible effect of ROCK inhibitor in mitophagy by destabilizing the actin cytoskeleton. This facilitates the formation of F-actin cages around depolarized mitochondria, which leads to their degradation. Moreover, the study speculates the effect attributed to increased levels of mitochondrial HK2 [14]. An additional study identified Hexokinase 1 (HK1) as a substrate that is ubiquitylated on decreasing mitochondrial membrane potential, by exogenous parkin, and strongly suggests HK1 as a substrate of Parkin. ROCK inhibitor is observed to increase HK2 activation and upregulate mitophagy [31].

This was evidenced in an additional screen performed, showing Parkin recruitment to depolarized mitochondria, identifying HK2 activity as required for this recruitment with shared effects of HK1. Further, knockdown of both HK1 and HK2 showed stronger inhibition of Parkin recruitment than HK2 alone. This explains that HK isoforms are required for Parkin relocation and targeting of mitochondria [29].

This provides us with facts that Hexokinase could be stated as an essential component in mitochondrial targeting and is a potential target for the treatment of Parkinson’s disease. This can be done by ROCK inhibition, which is shown to increase hexokinase activity.

Rho-associated kinase inhibitors

Several types of ROCK inhibitors have been reported. Fasudil (isoquinoline derivative) is one. It blocks ROCK competitively at the adenosine triphosphate (ATP) binding site of the kinase. Both ROCK1 and ROCKII are inhibited by fasudil with IC50 values of 0.26 μM and 0.32 μM, respectively. Hydroxyfasudil [32] and dimethylfasudil (H-1152P) are optimized derivatives of fasudil and demonstrate potency and selectivity [33].

Y-27632 is another type of ROCKI and ROCKII inhibitor that competitively binds to the ATP site [34]. A more potent ROCK inhibitor, i.e., Y-39983, exerts its effects on the axonal regeneration of crushed optic nerves [35].

Safety profile

ROCK inhibitor, Fasudil has not been evidenced to cause severe side effects on patients with cerebral vasospasm. Moreover, it was well-tolerated in clinical trials, including patients with conditions of stable angina or on the treatment of acute ischemic stroke. They showed no unsafe effects or adverse changes in heart rate and blood pressure readings [36].

Nevertheless, studies share that ROCK1 or ROCKII knockout mice demonstrated developmental irregularities, including extreme prenatal defects in mouse embryos on the administration of Y-27632, indicating less understood functions of ROCK during the developmental stages of the embryo. Further, the administration of Y-27632 exhibited behavioral changes in mice on emotional tests. More ocular side effects were seen on a high dosage of Y-39983 in eyes of rabbits and monkeys, showing punctate subconjunctival hemorrhages. The above-stated side effects in tested animals suggest that more in-depth assessments are required on ROCK inhibitors to have a better understanding of their safety profile [36].

Local effects on the eye include blepharitis and blurred vision. Systemically, ROCK inhibition was evidenced to induce an adjustable drop in lymphocyte counts in limited individuals [37].

Limitation

Studies conducted on the effect of ROCK inhibition on increasing hexokinase-mediated mitophagy by PINK1/Parkin process amplification specifically are recent. This article provides only details of 2 ROCK isoforms with little depth given to the effects of their inhibition individually.

## Conclusions

Evidenced studies elaborate on ROCK functions and provide ample suggestions on how its inhibition can make therapeutic contributions in the treatment of Parkinson’s disease. There are collective indications of increased dopaminergic activities in cells with inhibited ROCK and, similarly, dopaminergic deterioration when subjected to MPTP, supported by rising levels of ROCK noticed. Parkin relocation to damaged mitochondria and subsequent mitophagy is also indicated to be enhanced on ROCK inhibition, which causes an increase in HK2 activity: required for Parkin relocation, mediating mitochondrial engulfment.

These collective pieces of evidence are significant in establishing a cellular basis of Parkinson’s disease. It is essential that these molecular processes are thoroughly understood in order to create therapeutic advances in the near future. However, this was limited to insufficient studies on different isoforms of ROCK. Also, minor clarifications remain on the potential side effects of ROCK inhibition and is yet to be explored. Although ROCK inhibition has been shown to improve the process of axonal degeneration, functional recovery and remyelination remain unclear. The long-standing effects and properties of ROCK inhibition will have to be assessed, even though first preclinical long-term studies display adequate tolerability and safety. Further contributions to its side effect profile or its reassessment in translational trials in patients of PD could bring emerging conclusions about the impact of ROCK inhibitors and their efficacy and safety in neurodegenerative diseases.
